# Supplementary material for: Efficient nonlinear beam shaping in three-dimensional lithium niobate nonlinear photonic crystals
Source: Nat Commun. 2019 Sep 13;10:4193. doi: 10.1038/s41467-019-12251-0 (PMC6744429; doi:10.1038/s41467-019-12251-0)
Supplement: Supplementary file 1 — Supplementary information [file 41467_2019_12251_MOESM1_ESM.pdf]

Supplementary information for “Efficient nonlinear beam shaping in three-dimensional lithium niobate nonlinear photonic crystals”

Wei et al.

Supplementary note 1 | The design of three-dimensional (3D) $LiNbO_3$ NPCs.

Binary computer-generated hologram (CGH) theory was proposed by Lee, which records both the phase and amplitude information of an optical field[1]. Consider a laser-engineered two-dimensional (2D) NPC with amplitude-modulated $\chi^{(2)}$. For generating a SH beam of $E_{2\omega}$, propagating along the y-direction and polarizing along the z-direction, the structure function of the 2D NPC is given by[2]

$$\begin{aligned} f(x,z) &= T\left\{\cos\left[G_x x - \arg\left(E_{2\omega}\right)\right] - \cos\left[\sin^{-1}\operatorname{amp}\left(E_{2\omega}\right)\right]\right\} \\ &= \sum_{m=-\infty}^{\infty}\left[\frac{\sin\left(m\sin^{-1}\operatorname{amp}\left(E_{2\omega}\right)\right)}{\pi m}\right]\times e^{\mathrm{i}mG_x x}e^{-\mathrm{i}m\arg\left(E_{2\omega}\right)} \end{aligned} \quad (1)$$

where function T is defined as

$$T(X) = \begin{cases} 1, & X \geq 0 \\ 0, & X < 0 \end{cases}. \quad (2)$$

Here, G_x corresponds to the spatial frequency of a reference plane wave $e^{\mathrm{i}G_x x}$, the "arg" and "amp" functions donate the phase and amplitude of the target SH beam. Clearly, the target SH beam presents when $m = 1$.

In our 3D NPC, the additional dimension along the y-axis is periodically modulated to provide an extra longitudinal reciprocal vector to fulfill the QPM condition. The modulation function along the y-axis is $T\left[\cos\left(G_y y\right)\right]$ with a spatial frequency of G_y. Therefore, the structure function in our 3D NPC can be expressed as

$$\begin{aligned} f(x,y,z) &= T\left\{\cos\left[G_x x - \arg\left(E_{2\omega}\right)\right] - \cos\left[\sin^{-1}\operatorname{amp}\left(E_{2\omega}\right)\right]\right\}\times T\left[\cos\left(G_y y\right)\right] \\ &= \sum_{m,n=-\infty}^{\infty}\left[\frac{\sin\left(m\sin^{-1}\operatorname{amp}\left(E_{2\omega}\right)\right)\sin\left(n\pi/2\right)}{\pi^2 mn}\right]\times e^{\mathrm{i}\left(mG_x x+nG_y y\right)}e^{-\mathrm{i}m\arg\left(E_{2\omega}\right)} \end{aligned} \quad (3)$$

The target SH beam presents at the first diffraction order ($m = 1$). In addition, the phase term $e^{\mathrm{i}\left(mG_x x+nG_y y\right)}$ provide a reciprocal vector $m\mathbf{G}_x + n\mathbf{G}_y$ for complete QPM process.

Supplementary note 2 | Calculation of the theoretical QPM wavelengths.

One can deduce from Supplementary Equation (3) that the full QPM condition is written as

$$\left|\mathbf{k}_{2\omega} - 2\mathbf{k}_{\omega} - m\mathbf{G}_x - n\mathbf{G}_y\right| = 0. \quad (4)$$

where $\mathbf{G}_x$ and $\mathbf{G}_y$ represent the reciprocal vectors in the x-axis and y-axis, respectively. $\mathbf{k}_{\omega}$ and $\mathbf{k}_{2\omega}$ are the wavevectors of the fundamental and SH beam, respectively. In our experiment, the

first-order longitudinal reciprocal vector $\mathbf{G}_y$ (n = 1) and the first three orders of transvers reciprocal vectors $m\mathbf{G}_x$ (m = 1, 2, 3) are used. Considering that both the fundamental and SH beams polarize along the z-axis, the scalar form of Supplementary Equation (4) is

$$\left(\frac{4\pi n_{\omega}^{e}}{\lambda_{\omega}}+G_y\right)^2+\left(mG_x\right)^2-\left(\frac{2\pi n_{2\omega}^{e}}{\lambda_{2\omega}}\right)^2=0\,. \qquad (5)$$

The dispersion relationship of the Mg-doped $LiNbO_3$ crystal in our experiment is obtained from Ref. 3. In our 3D NPC, $G_x=G_y=2\pi/3\ \mu\mathrm{m}^{-1}$. From Supplementary Equation (5), the calculated QPM wavelengths are 819 nm, 801 nm, and 776 nm, which involve reciprocal vectors of $\mathbf{G}_x+\mathbf{G}_y$, $2\mathbf{G}_x+\mathbf{G}_y$, and $3\mathbf{G}_x+\mathbf{G}_y$, respectively.

Supplementary note 3 | Measurement of topological charge.

We use a cylindrical lens to measure the topological charges (TCs) of the generated SH vortex beams. The cylindrical lens is used to transform the vortex beams into the patterns as shown in Supplementary Figure 1. The TC is equal to the number of dark nodes between the bright fringes[4], which is counted to be 1, 2, and 3 for the 1st, 2nd, and 3rd diffraction orders, respectively.

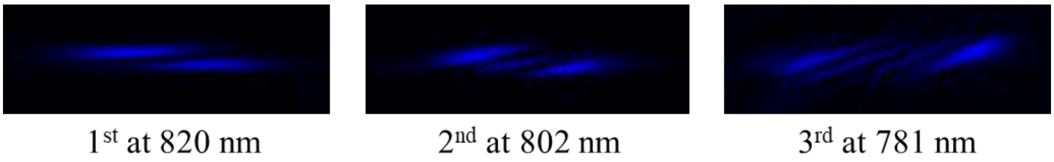

Supplementary Figure 1 The measured results of topological charges. By using a cylindrical lens, the converted patterns of the 1st, 2nd , and 3rd diffraction orders at their respective QPM wavelengths are shown from left to right.

Supplementary note 4 | Calculation of conversion efficiency.

For a SHG process in a NPC with dimension of $W\times H\times L$, the average SH power can be expressed as[5, 6]

$$\left\langle P_{2\omega}\right\rangle=\frac{2d_{\mathrm{eff}}^{2}L^{2}\omega^{2}\left\langle P_{\omega}\right\rangle^{2}t_{\mathrm{rep}}}{n_{\omega}^{2}n_{2\omega}\varepsilon_{0}c^{3}A_{\mathrm{eff}}\Delta t_{\mathrm{eff}}}\mathrm{sinc}^{2}\left(\frac{\Delta k_{x}W}{2}\right)\mathrm{sinc}^{2}\left(\frac{\Delta k_{y}H}{2}\right)\mathrm{sinc}^{2}\left(\frac{\Delta k_{z}L}{2}\right),\quad (6)$$

where d_{eff} is the effective nonlinear coefficient, $\left\langle P_{\omega}\right\rangle$ is average power of the fundamental beam,

A_{eff} is the effective area, Δt_{eff} is the pulse duration and t_{rep} is the repetition period. Group velocity dispersion is neglected considering that the interaction length is tens of microns in our experiment. Assume that the fundamental beam propagates along the *z*-axis. For nonlinear beam shaping, the transverse phase mismatch is generally compensated by the NPC structure, i.e., $\Delta k_x = \Delta k_y = 0$. Supplementary Equation (6) can be simplified as

$$\langle P_{2\omega}\rangle = \frac{2d_{\text{eff}}^2 L^2 \omega^2 \langle P_\omega\rangle^2 t_{\text{rep}}}{n_\omega^2 n_{2\omega}\varepsilon_0 c^3 A_{\text{eff}}\Delta t_{\text{eff}}}\text{sinc}^2\left(\frac{\Delta k_z L}{2}\right), \qquad (7)$$

Therefore, the conversion efficiency can be written as

$$\eta = \frac{\langle P_{2\omega}\rangle}{\langle P_\omega\rangle} = \frac{2d_{\text{eff}}^2 L^2 \omega^2 \langle P_\omega\rangle t_{\text{rep}}}{n_\omega^2 n_{2\omega}\varepsilon_0 c^3 A_{\text{eff}}\Delta t_{\text{eff}}}\text{sinc}^2\left(\frac{\Delta k_z L}{2}\right), \qquad (8)$$

while the normalized conversion efficiency is defined by

$$\eta_{\text{nor}} = \frac{\langle P_{2\omega}\rangle}{\langle P_\omega\rangle}\Big/\frac{\langle P_\omega\rangle t_{\text{rep}}}{\Delta t_{\text{eff}}} = \frac{2d_{\text{eff}}^2 L^2 \omega^2}{n_\omega^2 n_{2\omega}\varepsilon_0 c^3 A_{\text{eff}}}\text{sinc}^2\left(\frac{\Delta k_z L}{2}\right). \qquad (9)$$

Note that d_{eff} is directly related to spatial distribution of nonlinear coefficient

$$\chi^{(2)}(x,y,z) = d_{ij} - d_{ij}(1-v)f(x,y,z), \qquad (10).$$

where d_{ij} is the involve nonlinear coefficient, v is defined as the modulation depth, $f(x,y,z)$ is the structure function. In our experiment, v is about 85%, which is deduced from the measured SH conversion efficiencies. Performing Fourier transform of Supplementary Equation (10), one can get d_{eff}.

For nonlinear wavefront shaping in 2D NPC, the involved d_{ij} is d_{22}. Because $\Delta k_z \neq 0$, the normalized conversion efficiency can be written as

$$\eta_{\text{nor\_2D}} = \frac{2d_{\text{eff}}^2 \omega^2}{n_\omega^2 n_{2\omega}\varepsilon_0 c^3 A_{\text{eff}}}\frac{4\sin^2\left(\frac{\Delta k_z L}{2}\right)}{(\Delta k_z)^2}. \qquad (11)$$

One can see that the normalized conversion efficiency oscillates with the propagation length L.

For the 3D case, d_{ij} = d_{33}. In addition, full QPM condition can be satisfied, i.e. $\Delta k_x = \Delta k_y = \Delta k_z = 0$. The normalized conversion efficiency can be expressed as

$$\eta_{\text{nor\_3D}} = \frac{2d_{\text{eff\_3D}}^2 L^2 \omega^2}{n_\omega^2 n_{2\omega}\varepsilon_0 c^3 A_{\text{eff}}}, \qquad (12)$$

which increases quadratically with the length L.

Take the SH OAM modes of $l = 1$ for example. In our 3D NPC, $d_{eff\_3D} = 2.23\times10^{-13}$ m V^{-1}, L = 45 μm, $A_{eff} = 1600\pi$ μm^2. From Supplementary Equation (12), the theoretical normalized conversion efficiency is 3.1×10^{-10} W^{-1}. The measured value is 1.4×10^{-10} W^{-1}. Similarly, the theoretical normalized conversion efficiency of the SH HG(1,1) mode is calculated to be 2.7×10^{-9} W^{-1}, which is consistent with the measured value of 1.95×10^{-9} W^{-1}. The difference between the experimental and theoretical results could be attributed to the imperfect structure in 3D NPC.

The typical conversion efficiency of nonlinear beam shaping in 2D NPC[7] is 1×10^{-13} W^{-1}. Consider that the diameters of the fundamental beams are ~40 μm in our work and ~300 μm in Ref. 7. Under the same pump condition, the conversion efficiency of our 3D nonlinear beam shaping is enhanced by at least one order of magnitude in comparison to the 2D case.

Supplementary note 5 | Diffraction pattern of the fundamental wave.

The diffraction pattern of the fundamental wave in our 3D NPC sample is shown in Supplementary Figure 2. The modification of refractive index was measured to be about 5×10^{-3}. Because of the scattering and diffraction induced by the refractive index change, the conversion efficiency is decreased by a few percent in our experiment.

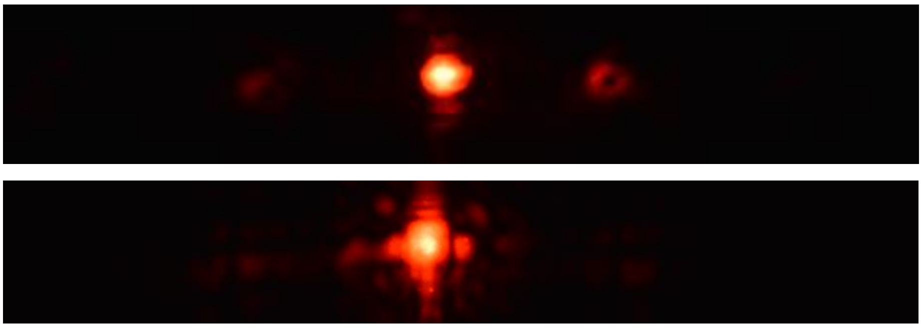

Supplementary Figure 2 Diffraction images of the fundamental wave through the 3D NPCs for OAM mode (up) and HG(1,1) mode (down).

Supplementary References

1 Lee, W.-H. Binary computer-generated holograms. *Appl. Opt.* **18**, 3661-3669 (1979).

2 Shapira, A., Shiloh, R., Juwiler, I. & Arie, A. Two-dimensional nonlinear beam shaping. *Opt. Lett.* **37**, 2136-2138 (2012).

3 Gayer, O., Sacks, Z., Galun, E. & Arie, A. Temperature and wavelength dependent refractive index equations for MgO-doped congruent and stoichiometric LiNbO3. *Appl. Phys. B* **91**, 343-348 (2008).

4 Fang, X. *et al.* Examining second-harmonic generation of high-order Laguerre-Gaussian modes

through a single cylindrical lens. *Opt. Lett.* **42**, 4387-4390 (2017).
5 Arie, A., Habshoosh, N. & Bahabad, A. Quasi phase matching in two-dimensional nonlinear photonic crystals. *Opt. Quant. Electron.* **39**, 361-375 (2007).
6 Weiner, A. M. Ultrafast Optics. (John Wiley & Sons, 2009).
7 Shapira, A., Shiloh, R., Juwiler, I. & Arie, A. Two-dimensional nonlinear beam shaping. *Opt. Lett.* **37**, 2136-2138 (2012).
